# Transforming non-conventional yeasts into key players in biotechnology: advances in synthetic biology applications

**DOI:** 10.3389/fmicb.2025.1600187

**Published:** 2025-05-02

**Authors:** Soo Young Moon, Nan-Yeong An, Ju Young Lee

**Affiliations:** ^1^Department of Biological Sciences, Korea Advanced Institute of Science and Technology (KAIST), Daejeon, Republic of Korea; ^2^Division of Interdisciplinary Bioscience and Bioengineering, Pohang University of Science and Technology (POSTECH), Pohang, Republic of Korea; ^3^Graduate School of Engineering Biology, Korea Advanced Institute of Science and Technology (KAIST), Daejeon, Republic of Korea

**Keywords:** non-conventional yeast, synthetic biology, gene editing tool, metabolic engineering, yeast biotechnology

## Abstract

Non-conventional yeasts exhibit exceptional genetic and functional diversity, serving as a largely untapped repertoire for biotechnological applications. Beyond the conventional yeast *Saccharomyces cerevisiae*, non-conventional yeasts are naturally more multifaceted, possessing the ability to utilize renewable and low-cost carbon sources while exhibiting robust physiology under challenging conditions. However, their vast potential remains largely unexplored, encompassing both challenges and opportunities for biotechnological advancements. Over the past decade, technological advancements in synthetic biology have unlocked new opportunities to harness their potential and overcome inherent limitations, enabling the full exploitation of their advantages across a broad spectrum of applications. In this review, we highlight recent advances in the synthetic biology of non-conventional yeasts, focusing on the development of new genetic building blocks (e.g., promoters and terminators), genome editing tools, and metabolic pathway engineering. Through these technologies, non-conventional yeasts are poised to emerge as pivotal next-generation workhorses tailored for specific applications in sustainable biomanufacturing, accelerating the transition to a bio-based economy.

## 1 Introduction

The convergence of environmental pollution, climate change, and resource scarcity is increasing intractable and compounding global challenges (Li W. et al., 2021; Karim et al., 2022; Liu et al., 2023). Microbial biotechnology provides a sustainable alternative (Raschmanova et al., 2018; Thorwall et al., 2020), enabling cost-effective and sustainable bioproduction across various medical, agricultural, food, and chemical industries. In particular, yeasts offer key advantages over other microbes in industrial biotechnology, including eukaryotic cellular machinery capable of post-translational modifications, the ability to utilize a wide range of inexpensive and renewable feedstocks, and robustness under harsh industrial conditions (Deparis et al., 2017; Nielsen, 2013; Qiu et al., 2019). Historically, yeasts have played crucial roles in the food industry and in the production of bulk and fine chemicals, as well as biofuels (Patra et al., 2021; Fabarius et al., 2021; Wang et al., 2025). Among them, *Saccharomyces cerevisiae* is the most extensively studied model yeast, known for its well-characterized genome and established molecular genetic engineering tools (Rainha et al., 2020; Moon et al., 2023). However, despite extensive research and engineering efforts, *S. cerevisiae* has inherent metabolic limitations, including low productivity, susceptibility to product toxicity, and an inability to convert alternative substrates into high-value products efficiently. These constraints restrict its commercial competitiveness and emphasize the need for alternative microbial platforms with superior metabolic capabilities (Park et al., 2022; Sibirny, 2023; Patra et al., 2021).

Potential solutions can be found in non-conventional yeasts, including *Yarrowia lipolytica*, *Pichia pastoris*, and *Kluyveromyces marxianus*. These yeasts exhibit superior metabolic flexibility, stress tolerance, and substrate utilization capabilities, making them highly attractive for industrial applications (Thorwall et al., 2020; Rebello et al., 2018; Wang et al., 2021; Nurcholis et al., 2020; Spohner et al., 2015; Yang and Zhang, 2018; Monteiro de Oliveira et al., 2021; Madhavan et al., 2017; Wagner and Alper, 2016). However, many non-conventional yeasts still suffer from limited genetic tractability, low transformation efficiency, and a lack of well-characterized regulatory parts, which constrain their broader application in biotechnology (Wagner and Alper, 2016; Lobs et al., 2017). In recent decades, the research on non-conventional yeasts has gained momentum, driven by rapid breakthroughs in synthetic biology. These advancements have significantly expanded their industrial utility by providing powerful tools for optimizing gene expression, metabolic pathways, and strain performance. This review covers a comprehensive overview of the attractive characteristics and role of non-conventional yeasts as versatile biotechnological workhorses, with a special emphasis on recent breakthroughs in synthetic biology that have enhanced their industrial applications. Key areas of focus include promoter and terminator engineering for precise gene regulation, CRISPR/Cas-based genome editing for efficient strain development, and pathway optimization strategies for improved biochemical production.

## 2 Non-conventional yeasts as biotechnological workhorses

**Figure 1:**
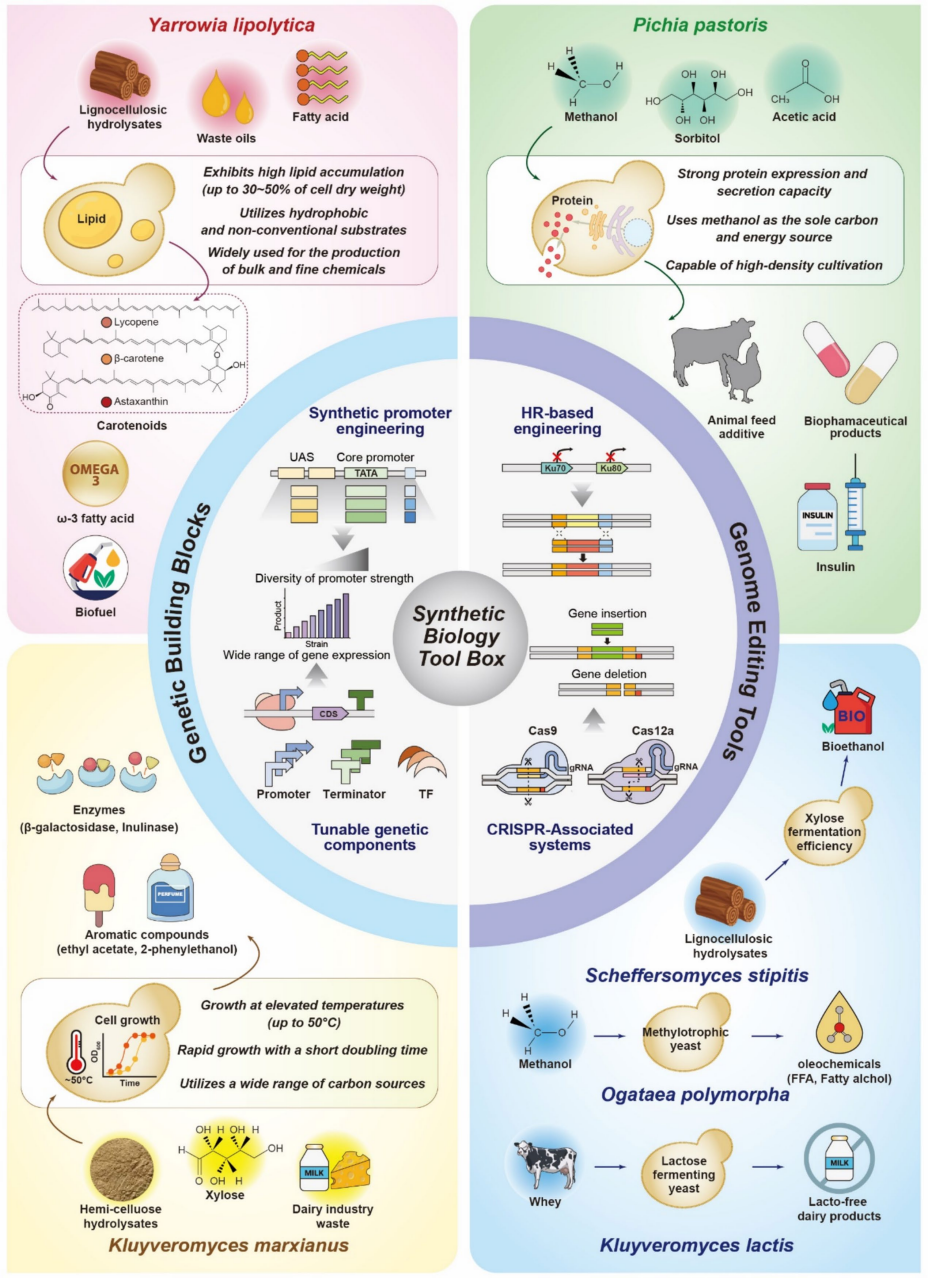
A key summary of synthetic biology tools and metabolic features of non-conventional yeasts for industrial biotechnology. This figure illustrates key synthetic biology strategies and the distinctive traits of non-conventional yeasts. Genetic components, including synthetic promoters, terminators, and transcription factors, enable fine-tuned control of gene expression. Genome editing tools such as homologous recombination via *KU70* or *KU80* deletion and CRISPR-associated systems (Cas9 and Cas12a) facilitate efficient and precise genome manipulation. Each non-conventional yeast exhibits unique metabolic traits suited for specific biotechnological uses. *Y. lipolytica* is characterized by high lipid accumulation and the ability to metabolize hydrophobic substrates, supporting the production of oleochemicals and carotenoids. *P. pastoris* exhibits strong protein expression and utilizes methanol and other cost-effective substrates, facilitating the industrial production of enzymes and pharmaceutical proteins. *K. marxianus* is known for thermotolerance, rapid growth, and broad substrate utilization, making it suitable for the biosynthesis of flavor compounds and industrial enzymes. *S. stipitis* efficiently ferments xylose derived from lignocellulosic biomass, contributing to bioethanol production. *O. polymorpha* metabolizes methanol to produce oleochemicals such as free fatty acids and fatty alcohols. *K. lactis* ferments lactose and is widely applied in dairy biotechnology. Collectively, these non-conventional yeasts offer complementary and adaptable platforms for sustainable and scalable bioproduction. UAS, upstream activating sequence; CDS, coding sequence; TF, transcription factor; HR, homologous recombination.

Non-conventional yeasts have emerged as new potential workhorses for the overproduction of fuels, chemicals, and pharmaceuticals owing to their robust physiology, which includes high tolerances to bioprocess-induced stresses (e.g., low pH, high temperatures, and osmolarity), resistance to inhibitory toxic compounds, and ability to utilize non-conventional feedstocks and synthesize large amounts of metabolites and proteins (Rebello et al., 2018; Thorwall et al., 2020; Wang et al., 2021; Markham and Alper, 2018; Spohner et al., 2015; Yang and Zhang, 2018; Nurcholis et al., 2020). Notably, the ability of non-conventional yeasts to metabolize non-conventional substrates—such as lignocellulosic hydrolysates, waste oils, and methanol—offers substantial industrial benefits. These substrates are often derived from low-cost, renewable sources like agricultural residues and CO₂-based industrial waste streams, thereby supporting more sustainable and economically viable biomanufacturing process (Rerop et al., 2023; Cotton et al., 2020; Do et al., 2019). For instance, methanol, a key substrate for *P. pastoris*, can be industrially produced from synthesis gas derived from natural gas or biomass, enabling cost-effective and potentially carbon-neutral feedstock supply chains (Cai et al., 2022). Among non-conventioal yeasts, *Y. lipolytica*, *P. pastoris* (recently reclassified as *Komagataella phaffii*), and *K. marxianus* are particularly notable for their distinct and inherent advantages in lipid accumulation, heterologous protein production, and thermotolerance, respectively. These species have been relatively well-characterized and are frequently used as biotechnological workhorses (Figure 1).

The oleaginous yeast *Y. lipolytica* is widely recognized for its exceptional lipid accumulation capacity, naturally reaching up to 30–50% of its cell dry weight, and its ability to metabolize diverse non-conventional substrates, including lignocellulosic hydrolysates, fatty acids, waste oils, crude glycerol, and acetate (Beopoulos et al., 2011; Madzak, 2021; Groenewald et al., 2014). Driven by efficient carbon flux through the acetyl-CoA and malonyl-CoA pathways, *Y. lipolytica* serves as a robust industrial chassis for commercial synthesis, supporting the production of lipids, advanced biofuels, and lipid-derived fine chemicals such as carotenoids and omega-3 fatty acids (Ma et al., 2020; Zhang et al., 2023; Xie et al., 2017; Shi and Zhao, 2017; Jiang et al., 2024; Ma et al., 2024; Liu Z. et al., 2024; Park et al., 2025; Sharpe et al., 2014). Additionally, its generally recognized as safe (GRAS) status makes *Y. lipolytica* a valuable platform for producing lipases widely used in the food, pharmaceutical, and environmental industries (Brígida et al., 2014; Hu et al., 2024).

*Pichia pastoris* is a methylotrophic yeast commonly used in the manufacture of industrial enzymes and pharmaceutical proteins owing to its various strengths. Notably, its strong protein expression and secretion capacity, along with its ability to perform post-translational modifications, make *P. pastoris* an ideal system for both cytosolic and secretory protein production (Gellissen et al., 2005; Karbalaei et al., 2020; Macauley-Patrick et al., 2005; Zha et al., 2023; Yang and Zhang, 2018). This feature enables the secretion of target proteins at high titers, simplifying downstream processing and facilitating the commercial production of pharmaceutical proteins, including human insulin and interferon, demonstrating the industrial relevance of this yeast (Patra et al., 2021; Nurdiani et al., 2024; Gao et al., 2021; Zha et al., 2023; Shrivastava et al., 2023). A further advantage of *P. pastoris* is its ability to utilize cost-effective substrates, tolerate high-stress conditions, and achieve higher cell densities than *S. cerevisiae*, making it a more suitable host for sustainable industrial processes. Owing to these attractive characteristics, *P. pastoris* has also been engineered to produce various value-added chemicals at low cultivation costs with high scalability (Lobs et al., 2017; Araya-Garay et al., 2012; Liu et al., 2015; Liu et al., 2018; Qian et al., 2022; Gao et al., 2023; Cai et al., 2022).

*Kluyveromyces marxianus* is also industrially relevant because of its beneficial traits, such as thermotolerance up to ~50°C, high growth rate, and broad substrate spectrum (e.g., hemi-cellulose hydrolysates, xylose, and dairy industry wastes) (Bilal et al., 2022; Lyu et al., 2021; Ha-Tran et al., 2020; Varela et al., 2017). Besides, *K. marxianus* serves as a natural producer of high-value ethyl acetate and other short-chain volatile esters, widely used as flavor and fragrance compounds (Lobs et al., 2018; Karim et al., 2020; Morrissey et al., 2015; Loser et al., 2013; Roy et al., 2023; Perpetuini et al., 2022). Its ability to grow at elevated temperatures facilitates simultaneous saccharification and fermentation of lignocellulosic and other polysaccharide-based feedstocks, reducing cooling costs, minimizing contamination risks, and improving bioprocess efficiency (Patra et al., 2021). Additionally, *K. marxianus* efficiently assimilates dairy byproducts such as lactose and cheese whey, taking a step toward more sustainable biomanufacturing (Bilal et al., 2022; Qiu et al., 2023).

Beyond these well-studied yeast species, several other non-conventional yeasts have recently attracted significant attention for their industrial potential. *K. lactis*, a GRAS yeast following *S. cerevisiae*, is a lactose-fermenting species frequently found in milk and dairy products. The *β*-galactosidase enzyme from *K. lactis*, which metabolizes milk constituents such as lactose, is widely used in the food industry to produce lactose-free dairy products (Becerra et al., 2001; Naumov et al., 2006). Furthermore, several metabolites are commercially produced in *K. lactis*, including lactate, D-gluconic acid derived from xylose, and D-arabitol produced from whey (Toivari et al., 2012; Toyoda and Ohtaguchi, 2011). While *P. pastoris* is primarily used for protein production, another methylotrophic yeast, *Ogataea polymorpha*, has attracted increasing attention as a promising chassis for producing various chemicals using methanol as the sole carbon source (Xie et al., 2024). *Scheffersomyces stipitis*, known for its superior xylose fermentation efficiency, holds promise for bioethanol production from lignocellulosic hydrolysates, as it converts xylose into ethanol with minimal or no xylitol byproduct formation, improving economic viability (Ruchala et al., 2020; Mastella et al., 2023; Kobayashi et al., 2022).

Still, beyond the natural advantages of these non-conventional yeasts, which make them more suited for tasks such as utilizing renewable and low-cost carbon sources or exhibiting high-stress tolerance, genetic engineering tailored to specific purposes can further enhance their functionality toward a robust bioeconomy in future biotechnological processes. To fully unlock the potential of these yeasts and facilitate their successful integration into biotechnological applications, genome engineering tools must be developed to pave the way for progress in metabolic engineering and synthetic biology to advance the application of new non-conventional yeasts rapidly. Below, we highlight recent synthetic biology tools and approaches that increase the industrial potential of non-conventional yeasts (Table 1). Key strategies include (1) promoter and terminator engineering for optimized gene expression and (2) CRISPR-based genome editing for efficient and multiplexed genetic modifications.

**Table 1:** Synthetic biology tools and their applications in non-conventional yeasts.

Synthetic biology tool	Description / Feature	Strain	Engineering strategy	Substrate	Products	Production titer (Scale)	Ref.
Promoter							
pMnDH2	Mannitol dehydrogenase promoter / 1.6-fold stronger than pTEF	*Y. lipolytica* Po1g	Overexpression of *at*UGT (UDP-glucosyltransferase)	Glucose	Salidroside	95.64 mg/L (250 mL Shake flask)	Wang et al. (2023)
pAOX1	Strong methanol-inducible promoter	*P. pastoris*	Co-expression of *Mit1* to enhance transcriptional activity	Methanol Glycerol	eGFP (Enhanced green fluorescent protein)	2.2-fold increase in fluorescence intensity (NR)	Haghighi Poodeh et al. (2022)
					VEGF (Vascular endothelial growth factor)	1.9-fold increase in secreted protein amount (NR)	
Hybrid promoter							
pMT-2-UAS16	Copper-inducible promoter enhanced with UAS16 for strong gene expression / 30-fold higher induction compared to native pMT-2	*Y. lipolytica* Po1f	Overexpression of codon-optimized *Mm*WS (wax ester synthase) gene	Glucose	Wax ester	149.5 mg/L (Shake flask)	Xiong and Chen (2020)
pTEF_R1_	Fatty acyl-CoA-responsive promoter / pTEF fused with bacterial transcriptional regulator FadR binding sites	*Y. lipolytica* Po1g *Ku70Δ*	Overexpression of Alk5 pTEF_R1_ enables fatty acyl-CoA-responsive expression of Alk5, allowing decoupling of growth and production phases.	Glucose	ω-hydroxy palmitic acid	160 mg/L (1 L batch fermentation)	Park et al. (2021)
pUAS1B4-EXPm	Strong promoter combining 4 copies of yeast UAS with the EXPm core promoter	*Y. lipolytica* Po1g *Ku70Δ*	Overexpression of *Sc*ARO10	Glucose	Isoamyl alcohol	11.57 mg/L (250 mL Shake flask)	Zhao et al. (2021)
pUAS1B4-LEUm	Moderate-strength promoter combining 4 copies of strong yeast UAS with LEUm core promoter		Overexpression of *Sc*BAT1 and *Sc*ADH2				
pKmIMTCP2	Constitutive promoter / uncharacterized cell wall protein promoter	*K. marxianus* NBRC1777	Overexpression of *lacZ* (β-galactosidase) from *K. marxianus*	Dextrose, xylose	β-galactosidase	1800 Miller Units	Kumar et al. (2021)
pAOX1	Strongest methanol-inducible promoter / pAOX1 promoter combined with K4 Kozak sequence	*P. pastoris* GS115	Overexpression of C4ST	Methanol Glycerol	Chondroitin sulfate A	182.0 mg/L (Shake flask), 2.1 g/L (3 L bioreactor)	Jin et al. (2021)
cTRDL (constitutive Transcriptional Device Library)	126 constitutive promoter variants with expression levels ranging from 16% to 520% relative to pAOX1	*P. pastoris* GS115	Overexpression of s*LovA* (codon-optimized *LovA*, cytochrome P450 monooxygenase), and CPR (cytochrome P450 oxidoreductase)	Methanol	Monacolin J	208 mg/L (250 mL Shake flask)	Zhu et al. (2022)
iTRDL (inducible Transcriptional Device Library)	162 methanol-inducible promoter variants with expression levels ranging from 30% to 500% relative to pAOX1	*P. pastoris* GS115	Overexpression of *LovB* (nonaketide synthase), *LovC* (enoyl reductase), *LovG* (thioesterase), and *NpgA* (phosphopantetheinyl transferase)	Methanol	Dihydromonacolin L	250 mg/L (250 mL Shake flask)	Zhu et al. (2022)
pSNT5	Engineered ADH2-derived promoters combining UAS elements and removing URS to enhance expression	*P. pastoris* GS115	Overexpression of *XylB* (xylanase)	Glycerol	Xylanase	2.2-fold increase compared to the native pADH2 (5 L bioreactor)	Erden-Karaoglan et al. (2022)
phy47-7	pGAP1-based hybrid promoter incorporating regulatory elements from KAR2 and GCW14 to enhance transcriptional activity	*P. pastoris* GS115	Overexpression of *PS* (α-pinene synthase)	Glucose	Pinene	1.18 mg/L (NR)	Lai et al. (2024)
				Glycerol		2.20 mg/L (NR)	
pIN450	Hybrid promoter combining regulatory elements of the carbon-responsive ICL1 promoter with the strong constitutive NC1 promoter from *K. marxianus*	*K. marxianus* CBS712Δ*U*	Overexpression of 2-PS (2-pyrone synthase)	Lactose	Triacetic acid lactone	1.39 g/L (3 mL tube)	Bassett and Da Silva (2024)
			Overexpression of 6-MSAS (6-methylsalicylic acid synthase) and *npgA* (4′-phosphopantetheinyl transferase)	Lactose	6-Methylsalicylic acid	1.09 g/L (3 mL tube)	
			Overexpression of *IaaM* (tryptophan-2-monooxygenase) and *IaaH* (indole-3-acetamide hydrolase)	Lactose tryptophan	Indole-3-acetic acid	3.6-fold increase compared to pNC1 (3 mL tube)	
			Overexpression of *SabS1* (sabinene synthase)	Xylose	Sabinene	1.5 mg/L (3 mL tube)	
Terminator							
XPR2t	Native terminator from *Y. lipolytica*, commonly used for heterologous expression	*Y. lipolytica* Po1g	Overexpression of prorennin (prochymosin A allele)	Sucrose	Prorennin	160 mg/L (5 L batch fermentation)	Madzak et al. (2000)
DHASt	Native terminator from *P. pastoris* / High-expression gene terminator from methanol utilization pathway, enhancing mRNA stability	*P. pastoris* X-33	Overexpression of *CalB*	Glucose	Lipase	3-fold increase compared to AOX1t under pAOX1 (NR)	Ramakrishnan et al. (2020)
AOX1t	Native strongest terminator in *P. pastoris*, providing mRNA stability and high protein expression	*P. pastoris* CBS7435	Overexpression of EGFP	Glycerol	EGFP	17-fold increase compared to *Sc*GIC1t under pGAPDH (NR)	Ito et al. (2020)
			Overexpression of *β*-Glucosidase from *Aspergillus aculeatus*	Glycerol	β-Glucosidase	3.6-fold increase compared to *Sc*ICY2t under pGAPDH (NR)	
			Overexpression of CYP76AD1 (W13L/F309L) and DOD (DOPA deoxygenase)	Glycerol	Betaxanthin	8.36-fold increase compared to *Sc*ICY2t under pGAPDH (NR)	
CRISPR tool							
CRISPR/Cas9	Disruption rates of PEX10 (86%) and MFE1 (100%)	*Y. lipolytica* Po1f	Increased HR efficiency through *KU70* deletion	Glucose, Oleic acid	NR	NR	Schwartz et al. (2016)
nickase Cas9	Multiplex gene disruption of *TRP1, PEX10,* and *HIS3*: 94% (single), 31% (double)	*Y. lipolytica* Po1g *ku70*Δ	Target-AID (activation-induced cytidine deaminase) system for introducing a nonsense mutation	Glucose, Oleic acid	NR	NR	Bae et al. (2020)
Cas12a/Cpf1	Editing efficiencies of up to 96% for counter-selectable markers (*CAN1*, *URA3*) and up to 80% for auxotrophic markers (*MET2, MET25, MET6*)	*Y. lipolytica* Po1g	Optimized crRNA expression and polyU modifications for precise and multiplexed genome editing	Dextrose	NR	NR	Yang et al. (2020)
CRISPR/Cas9	Editing efficiency above 80% for base insertions, deletions, and a single-point mutation	*P. pastoris* GS115	Site-specific deletion, insertion, or substitution of the S215 residue of transcriptional activator MXR1 on the chromosomes	Methanol	NR	NR	Hou et al. (2020)
CRISPR/Cas9	One-step integration of a three-gene expression cassette into a single genomic locus (~60% efficiency)	*K. marxianus* CBS 6556	Multigene integration of shikimate pathway (*Km*ARO4K221L, *Km*PHA2, and *Km*ARO7G141S)	Glucose	2-phenylethanol	1,943 mg/L (250 mL Shake flask)	Li M. et al. (2021)
CRISPR/Cas9	Gene editing efficiency not reported	*K. lactis* GG799	Knocked out *INV* (endogenous invertase) to improve fructosyltransferase activity	Glucose Galactose	Fructo-oligosaccharide	NR	Burghardt et al. (2020)
CRISPR/Cas9	Gene editing efficiency not reported	*C. tropicalis* CU-208	Gene editing of key pathway enzymes t*CBTS1* (truncated Cembratriene-ol Synthase 1), *ERG20* (Farnesyl Pyrophosphate Synthase), *BTS1* (Geranylgeranyl diphosphate Synthase) Enhanced expression of *ERG20* and *BTS1* under the strong pGAP1	Glucose	Cembratriene-ol	1,425.76 mg/L (NR)	Zhang et al. (2024)
CRISPR-assisted Cre recombination	Iterative genome editing using CRISPR-SpCas9 and Cre-loxP system	*R. toruloides* RT1389	Integrating EGT biosynthetic genes (*Egt1* and *Egt2*) and optimizing the S-adenosylmethionine pathway	Glucose Xylose	Ergothioneine (EGT)	267.4 mg/L (NR)	Liu K. et al. (2024)

NR, Not Reported.

## 3 Synthetic biology tools and approaches to unlock the potential and function of non-conventional yeasts

### 3.1 Genetic building blocks for synthetic pathway engineering: promoters and terminators

The advancement and implementation of synthetic biology tools, combined with the expanding library of genetic building blocks, have significantly increased the utility of non-conventional yeasts as versatile systems and chassis cells in biotechnological applications. Recent studies have driven significant progress in their application, leveraging the availability of genetic elements such as promoters and terminators specifically tailored to non-conventional yeasts (Ji et al., 2024; Patra et al., 2021; Ma et al., 2020; Gao et al., 2021; Wang et al., 2023; Kumar et al., 2021; Teo and Chang, 2014; Qiu et al., 2023). These genetic elements play crucial roles in regulating transcription rates and mRNA stability, directly influencing protein expression levels and consequently enabling non-conventional yeasts to emerge as valuable platforms for synthetic biology and biomanufacturing (Patra et al., 2021; Ito et al., 2020; Wagner and Alper, 2016; Sun et al., 2022) (Figure 1).

#### 3.1.1 Promoters

Promoters, in particular, are critical determinants of transcriptional regulation, as they govern the timing, strength, and spatial patterns of gene expression. This, in turn, profoundly shapes metabolic activities, enabling the precise modulation of cellular behaviors (Blazeck and Alper, 2013; Ji et al., 2024; Ma et al., 2020; Wang et al., 2023). Hence, discovering and selecting appropriate promoters, as well as engineering novel promoter elements, are fundamental steps in advancing metabolic engineering and synthetic biology for non-conventional yeasts (Madhavan et al., 2017; Rebello et al., 2018). Generally, a significant strategy for achieving high-expression of a given protein involved in synthetic pathways is using a strong and constitutive promoter. Strong promoters are typically derived from genes associated with the essential functions or unique metabolic traits of each yeast species. Representative examples include promoters driving translation (e.g., pTEF active across multiple yeast species), methanol utilization (e.g., pAOX1 in *P. pastoris*), or ethanol utilization (e.g., pADH2 in *S. cerevisiae*), taking advantage of the inherent metabolic capabilities and physiological traits of the respective species of yeast.

Among these promoters, the endogenous translation elongation factor-1α promoter (pTEF) is the most widely used because of its robust constitutive expression across diverse yeast species (Gu et al., 2023; Larroude et al., 2018; Ahn et al., 2007; Steiner and Philippsen, 1994; Kitamoto et al., 1998). In addition to the TEF1 promoter, to expand regulatory options in *Y. lipolytica*, a library of 81 endogenous promoters, primarily associated with carbon and nitrogen metabolism, has been systematically screened, offering expression strengths ranging from 0.0006- to 1.60-fold relative to pTEF. Notably, the MnDH2 promoter (encoding mannitol dehydrogenase) exhibited the highest strength, achieving an expression level 1.60-fold greater than pTEF. This promoter facilitated the production of the plant-derived aromatic compound salidroside in *Y. lipolytica*, reaching a titer of 95.64 mg/L, the highest reported to date (Wang et al., 2023).

Alternatively, artificial hybrid promoters, combining upstream activation sequences (UASs) with modified core promoter elements, have been developed to enhance gene expression control in *Y. lipolytica*, providing greater flexibility and dynamic regulation (Blazeck et al., 2011). A notable example is the development of fatty acid-sensitive hybrid promoters by combining pTEF with bacterial transcriptional regulator FadR binding sites. Fatty acyl-CoA binds to FadR, inducing a conformational change that inhibits FadR binding to its target sequences, thereby upregulating the expression of target genes. This mechanism allows FadR hybrid promoters to decouple the cell growth and production phases in response to intracellular fatty acyl-CoA concentrations, producing 160 mg/L of *ω*-hydroxy palmitic acid (Park et al., 2021).

Hybrid promoters incorporating UAS elements have also addressed the limited number of native *Y. lipolytica* promoters available, significantly expanding expression flexibility. For instance, a hybrid promoter incorporating sixteen copies of the UAS from the *Y. lipolytica* alkaline extracellular protease promoter into the copper-inducible MT-2 core promoter facilitated the efficient production of wax esters at a titer of 149.5 mg/L (Blazeck et al., 2011; Xiong and Chen, 2020). Similarly, another hybrid promoter, which incorporates four copies of the *Y. lipolytica* alkaline extracellular protease promoter UAS with the export protein EXP1 promoter, achieved an isoamyl alcohol titer of 11.57 mg/L (Zhao et al., 2021). These advancements highlight the versatility of hybrid promoters in *Y. lipolytica*, enabling the production of diverse valuable compounds and optimizing cell factory applications.

In the methylotrophic yeast *P. pastoris*, promoter engineering has advanced with the AOX1 promoter, a highly active methanol-inducible promoter widely used for recombinant protein production (Wu et al., 2023). To create pAOX1 variants with variable strengths, Zhu et al. (2022) fused bacterial DNA-binding proteins with yeast transactivation domains and linked bacterial binding sequences to the AOX1 core promoter. Consequently, 126 constitutive hybrid promoter libraries with expression strengths ranging from 16% to 520% and 162 methanol-inducible hybrid promoter libraries ranging from 30% to 500% were constructed relative to the native AOX1 promoter (Zhu et al., 2022).

In addition, in a recent study, various Kozak sequences were applied to the AOX1 promoter to enhance the intracellular expression of chondroitin-4-O-sulfotransferase (C4ST), a membrane-bound enzyme rarely expressed in microorganisms. Combined with chondroitin biosynthesis pathway genes, hybrid promoter-driven C4ST expression produced 182.0 mg/L of chondroitin sulfate A in *P. pastoris* (Jin et al., 2021). In another effort to improve the AOX1 promoter efficiency, the overexpression of methanol-induced transcription factor 1 (*Mit1*) strongly activated the AOX1 promoter and increased eGFP production by 2.2-fold. Doubling the methanol feed concentration further boosted the eGFP output by an additional 1.3-fold (Haghighi Poodeh et al., 2022). Similar to the activation of pAOX1 mediated by *Mit1* overexpression, a separate study was dedicated to modifying the alcohol dehydrogenase 2 promoter (pADH2) based on its transcriptional regulatory mechanism. By replacing its repressor region with an activator region, pADH2 activity was enhanced by 2.2-fold compared with that of original pADH2 (Erden-Karaoglan et al., 2022).

Besides pAOX1, the glyceraldehyde-3-phosphate dehydrogenase promoter (pGAP) is commonly used as a constitutive promoter for protein expression in *P. pastoris*. Unlike pAOX1, pGAP does not require a toxic methanol inducer, making it suitable for continuous cultivation while maintaining stable cellular function (Wu et al., 2023; Vogl et al., 2016). Lai et al. (2024) developed a novel randomized hybrid promoter library derived from pGAP1 and demonstrated its potential by producing 1.18 mg/L of the biologically active natural monoterpene pinene, representing an 18% increase over the native GAP promoter.

Recent studies on *K. marxianus* have also been focused on identifying and optimizing native-derived promoters, from the weakest promoter REV1 (deoxycytidyl transferase) to the strongest promoter PDC1 (pyruvate decarboxylase), enabling 40-fold variation in gene expression (Qiu et al., 2023; Rajkumar et al., 2019). Among these efforts, novel expression toolkits were constructed by combining various promoters and terminators derived from *K. marxianus*. For example, p*Km*IMTCP2-*Km*IMTT1t, comprising an uncharacterized cell wall protein promoter (pIMTCP2) and a maltose transporter terminator (IMTT1t), demonstrated the highest activity in *K. marxianus*, producing approximately 1800 Miller units of *β*-galactosidase (Kumar et al., 2021).

Most recently, Bassett and Da Silva (2024) designed and built a novel carbon-responsive hybrid promoter, pIN450, by combining regulatory elements of the native *K. marxianus* carbon-responsive ICL1 promoter with the strong constitutive NC1 promoter from *K. marxianus*. The hybrid IN450 promoter exhibits carbon-responsive behavior in lactose and constitutive behavior in xylose, leading to over a 50% increase in the production of the high-value chemical triacetic acid lactone and a 6.6-fold increase in the production of the fungal polyketide 6-methylsalicylic acid compared to native pICL1 (Bassett and Da Silva, 2024).

#### 3.1.2 Terminators

Terminators are also essential in transcriptional regulation, influencing mRNA stability, half-life, and abundance, directly affecting protein expression levels (Gu et al., 2023; Patra et al., 2021; Hu et al., 2024; Madzak, 2021). Despite their vital roles, only a few terminators in non-conventional yeasts have been systematically characterized. Meanwhile, several *S. cerevisiae* terminators have been successfully adapted to non-conventional yeasts, such as *Sc*CYC1t in *Y. lipolytica* and *P. pastoris*, *Sc*ADH1t and *Sc*PGK1t in *K. marxianus*, and *Sc*ADH1t in *H. polymorpha* (Madzak, 2021; Patra et al., 2021). Native and synthetic terminators from non-conventional yeasts have also demonstrated potential in modulating gene expression (Table 1). In *Y. lipolytica*, native terminators such as XPR2t (extracellular protease), LIP2t (extracellular lipase), and CyC1t (cytochrome C) have been identified and characterized (Madzak et al., 2000; Ma et al., 2020). In *P. pastoris*, DHASt (dihydroxyacetone synthase) enhanced the expression of *Candida antarctica* lipase B (*CalB*) by 3-fold compared with AOX1t under pAOX1 (Ramakrishnan et al., 2020). Additionally, a library of 72 terminators from *S. cerevisiae*, *P. pastoris*, and synthetic sources demonstrated a 17-fold tunable range of activity in *P. pastoris* (Ito et al., 2020). The IMTT1 (*IMTCP1*) and IMTT2 (*IMTCP2*) terminators from *K. marxianus* significantly increased β-galactosidase production (Kumar et al., 2021), highlighting the versatility of terminator engineering for optimizing gene expression (Ito et al., 2020).

### 3.2 Genome editing tool: CRISPR-based genome editing

Efficient genome editing tools for inserting, deleting, and altering target genes are critical for engineering non-conventional yeasts to reconstruct complex metabolism and thus enhance product synthesis for industrial applications. Homologous recombination (HR)-mediated tools are generally preferred in genetic engineering due to their ability to precisely control integration loci, minimizing the risk of disrupting essential genes (Flagfeldt et al., 2009; Cai et al., 2019; Donohoue et al., 2018; Xia et al., 2023). However, unlike *S. cerevisiae*, non-conventional yeasts face unique challenges because non-homologous end joining (NHEJ) dominates over HR, which often leads to imprecise integration of inserted DNA (Xia et al., 2023; Cai et al., 2019). Overcoming the natural dominance of NHEJ to increase HR efficiency in non-conventional yeasts remains a significant challenge. Nonetheless, modulation of the NHEJ or HR DNA repair pathways has shown promise in addressing this issue. For instance, deletion of native NHEJ-promoting genes such as *Ku70* or *Ku80* significantly increased HR efficiency in many non-conventional yeasts (Maassen et al., 2008; Kooistra et al., 2004; Verbeke et al., 2013; Naatsaari et al., 2012; Saraya et al., 2012; Choo et al., 2014). Furthermore, the overexpression of HR repair proteins such as RAD52, RAD59, MRE11, and SAE2 from *S. cerevisiae* has achieved multiplex gene integration efficiencies of 100%, ~98%, and ~81% at single, double, and triple loci, respectively, even with homology arms as short as 40 bp (Gao et al., 2022).

The advent of CRISPR/Cas9 technology has revolutionized genome editing in non-conventional yeasts, offering unparalleled precision, flexibility, multiplexing, and simplicity (Bai et al., 2023) (Figure 1, Table 1). The CRISPR/Cas9-mediated genome editing tool introduces targeted double-strand breaks (DSBs) at specific loci, enabling precise and programmable modifications guided by customized simple single-guide RNA (sgRNA), with the assistance of intracellular DNA repair pathways such as HR and NHEJ. CRISPR/Cas9 streamlines the editing workflow, improves accuracy, and accelerates strain engineering to achieve desired properties (Zha et al., 2023; Wu et al., 2023; Schwartz et al., 2016; Li M. et al., 2021; Burghardt et al., 2020; Zhang et al., 2024; Liu K. et al., 2024).

In *Y. lipolytica*, CRISPR/Cas9 was first adapted in 2016 using a codon-optimized Cas9 and sgRNA expression under a synthetic RNA polymerase III promoter to disrupt genes such as *Ku70*, as well as lipid oxidation-related *Pex10* and *Mfe1* (Schwartz et al., 2016). More recently, a base editor combining CRISPR/Cas9, cytidine deaminase, and uracil glycosylase inhibitor enabled targeted base modifications without introducing DSBs, further expanding the genome editing toolbox in *Y. lipolytica*. This system achieved editing efficiencies of 94% for single genes and 34% for dual genes, demonstrating its potential for precise genetic engineering (Bae et al., 2020). Additionally, the CRISPR-Cas12a/Cpf1 system has been implemented, allowing for the retention of PAM sites after NHEJ repair and enabling efficient multiplexed editing (Yang et al., 2020). Using this system, the single-gene disruption efficiencies reached 99%, while triplex edits achieved up to 30%, highlighting its utility for complex genetic modifications.

In *P. pastoris*, a highly efficient CRISPR/Cas9 system was developed through the systematic optimization of codon-optimized Cas9 DNA sequences, various sgRNA sequences, and promoters for the optimal expression of both Cas9 and sgRNA, achieving genome editing efficiencies approaching 100% (Weninger et al., 2016). This system enabled targeted editing of the methanol expression regulator MXR1 and facilitated base insertions and deletions at critical amino acid positions, allowing for studying this transcription factor and its targets (Hou et al., 2020). Additionally, the CRISPR/Cas12a system enabled the deletion of large DNA fragments (up to 20 kb) and one-step integration of multiplexed genes, exhibiting 99% efficiency for single-gene edits, 65–80% efficiency for duplex edits, and 30% efficiency for triplex integrations (Zhang et al., 2021).

CRISPR/Cas9 has also been applied in *Kluyveromyces* species, demonstrating its versatility for gene deletion and multiplexed gene integration. In *K. marxianus*, a CRISPR/Cas9-based multigene integration system was developed to engineer key genes in the shikimate pathway (*Km*ARO4K221L, *Km*PHA2, and *Km*ARO7G141S), resulting in a 2.8-fold increase in the production of the rose-scented flavor and fragrance compound 2-phenylethanol. Further optimization of the Ehrlich pathway through the overexpression of ARO10 and inactivation of EAT1 boosted 2-phenylethanol production to 1,943 ± 63 mg/L under fed-batch conditions (Li M. et al., 2021). In *K. lactis*, CRISPR/Cas9 was successfully applied to delete the endogenous invertase gene, resulting in a 66.9% increase in fructose transferase activity (Burghardt et al., 2020).

Similar advances have also been achieved in other non-conventional yeasts. In *C. tropicalis*, CRISPR/Cas9 enhanced the production of the plant-derived macrocyclic diterpene cembratriene-ol to 1,425.76 mg/L, a 1,602-fold increase, by integrating the codon-optimized cembratriene-ol synthase gene and optimizing metabolic flux (Zhang et al., 2024). Likewise, in *Rhodotorula toruloides*, the CRISPR-assisted Cre recombination system, which combines CRISPR/Cas9 with the site-specific recombinase Cre, enables iterative genome editing. This approach increased the production of ergothioneine (EGT), a high-value antioxidant and cytoprotectant, to 267.4 mg/L, a 1.5-fold improvement, by integrating EGT biosynthetic genes (*Egt1* and *Egt2*) and optimizing the S-adenosylmethionine pathway (Liu K. et al., 2024).

## 4 Conclusions and future perspectives

Technological developments in synthetic biology, particularly in transcriptional regulation systems, CRISPR-based genome editing, and strain engineering, have greatly expanded the potential of non-conventional yeasts while overcoming their inherent limitations. Their applications are diverse, and they likely represent a crucial new means to address the looming challenges of biomanufacturing—for both narrow and broad product ranges—by enabling the production of a wide array of bio-based chemicals, fuels, and materials, thereby positioning them as valuable assets in the future of industrial biotechnology. In the near future, emerging technologies such as the design-build-test-learn cycle in synthetic biology will further accelerate this progress, driving the development of advanced and cost-effective methods for building, editing, and screening non-conventional yeasts with novel and optimized functions in a high-throughput manner, solidifying them as next-generation microbial workhorses for industrial biotechnology (Li X. et al., 2023; Whitford et al., 2021; Moon et al., 2024; Son et al., 2024).

The integration of automated genome synthesis, AI-assisted metabolic design, and omics-driven pathway optimization will further strengthen the potential of non-conventional yeasts, enabling precise metabolic fine-tuning for a wide range of applications (Patra et al., 2021; Darvishi et al., 2021; Li M. et al., 2023; Madhavan et al., 2017; Wang et al., 2025). Looking ahead, the convergence of synthetic biology, systems biology, and machine learning will be instrumental in streamlining strain engineering workflows, improving predictive modeling accuracy, and enhancing strain design efficiency. Additionally, expanding the molecular toolbox for non-conventional yeasts—including novel inducible promoters, tunable gene circuits, and genome-scale engineering strategies—will further enhance their versatility and adaptability. To fully realize the potential of non-conventional yeasts in biomanufacturing, future advances in synthetic biology should focus on addressing current limitations—such as the limited availability of species-specific regulatory elements and the narrow range of inducible promoters. Expanding modular, programmable, and scalable toolkits will enable more precise, flexible, and context-specific strain engineering, thereby accelerating the transition toward next-generation yeast-based production systems.

As synthetic biology continues to bridge the gap between a detailed understanding of non-conventional yeasts and their practical applications, these yeasts will undoubtedly play an expanding role in the global bioeconomy. Future research will likely be focused on harnessing automation, leveraging AI-driven metabolic design, and integrating multi-omics datasets to drive innovation in yeast engineering. Non-conventional yeasts will not only provide additional options alongside existing microbial platforms but also offer unique and tailored solutions for sustainable bioproduction, accelerating the transition toward a more sustainable bioeconomy.
